# CDK-Dependent Nuclear Localization of B-Cyclin Clb1 Promotes FEAR Activation during Meiosis I in Budding Yeast

**DOI:** 10.1371/journal.pone.0079001

**Published:** 2013-11-01

**Authors:** Katherine Louise Tibbles, Sourav Sarkar, Bela Novak, Prakash Arumugam

**Affiliations:** 1 University of Warwick, Coventry, United Kingdom; 2 Department of Biochemistry, University of Oxford, Oxford, United Kingdom; Florida State University, United States of America

## Abstract

Cyclin-dependent kinases (CDK) are master regulators of the cell cycle in eukaryotes. CDK activity is regulated by the presence, post-translational modification and spatial localization of its regulatory subunit cyclin. In budding yeast, the B-cyclin Clb1 is phosphorylated and localizes to the nucleus during meiosis I. However the functional significance of Clb1's phosphorylation and nuclear localization and their mutual dependency is unknown. In this paper, we demonstrate that meiosis-specific phosphorylation of Clb1 requires its import to the nucleus but not *vice versa*. While Clb1 phosphorylation is dependent on activity of both CDK and polo-like kinase Cdc5, its nuclear localization requires CDK but not Cdc5 activity. Furthermore we show that increased nuclear localization of Clb1 during meiosis enhances activation of FEAR (Cdc Fourteen Early Anaphase Release) pathway. We discuss the significance of our results in relation to regulation of exit from meiosis I.

## Introduction

Meiosis is a specialised form of cell division required for sexual reproduction, in which a diploid cell divides to form four haploid gametes. Two nuclear divisions must be achieved without an intervening round of DNA replication, in order to halve chromosome number [1], [2]. This is distinct from the mitotic cell cycle, in which the cell strictly alternates DNA replication and nuclear division in order to maintain chromosome number.

Alternation of DNA replication and nuclear division during the mitotic cycle is achieved by oscillations of Cyclin-Dependent Kinase (CDK) activity. As CDK activity rises following exit from G1, DNA replication is initiated. While CDK promotes initiation of DNA replication it also prevents re-initiation of DNA replication by phosphorylating components of the replication machinery [3], [4]. Further increase in CDK activity results in entry into M-phase, formation of a metaphase spindle and preparation for nuclear division. Destruction of sister chromatid cohesion by a cysteine protease, separase, triggers the movement of chromosomes towards opposite spindle poles of the cell, referred to as the metaphase-anaphase transition. A sharp drop in CDK activity following anaphase allows cells to exit from mitosis and undergo cytokinesis. The drop in CDK activity also permits the relicensing of DNA replication origins, a requirement for the next round of DNA replication [4]. Thus co-regulation of DNA replication and nuclear division by oscillation of CDK activity ensures their strict alternation.

Dephosphorylation of CDK substrates and the precipitous drop in CDK activity during anaphase in budding yeast are caused by the release of CDK-antagonising phosphatase Cdc14 [5]. Cdc14 spends most of the cell cycle in the nucleolus bound to its inhibitor Net1 [6]. During mitotic exit, its release occurs under the control of two regulatory networks, the Cdc14 Early Anaphase Release Network (FEAR), and the Mitotic Exit Network (MEN) [7], [8], which act to increase the phosphorylation of Net1 and reduce its ability to bind Cdc14 [6], [8]. During metaphase, phosphorylation of Net1 by CDK [9] is countered by the protein phosphatase PP2A^Cdc55^ [10], [11]. During anaphase, activated separase inhibits PP2A^Cdc55^ through a poorly understood mechanism. This results in increased Net1 phosphorylation by CDK [12] and release of Cdc14 from the nucleolus referred to as FEAR. Phosphorylation of the nucleolar protein Spo12 by CDK also leads to reduction of Net1-Cdc14 interaction [13], [14]. After being activated by CDK, the polo kinase Cdc5 promotes FEAR activation by inducing the degradation of Swe1, which is an inhibitor of mitotic CDK but not S-phase CDK [15]. In addition, Cdc5 also triggers MEN activation by phosphorylating Bfa1, a negative regulator of the MEN pathway [16]. The nucleolar release of Cdc14 triggers activation of kinases (Cdc15 and Dbf2/Dbf20) that phosphorylate both Net1 and Cdc14 [17], [18] independently of CDK activity. This causes sustained release of Cdc14 and completes mitotic exit.

The FEAR network-driven Cdc14 release is necessarily transient, as the FEAR network relies on CDK activity to maintain Cdc14 release. As the released phosphatase counters CDK activity, this causes its own resequestration into the nucleolus. MEN mutants, in which Cdc14 release is driven only by the FEAR network, arrest in anaphase with intact spindles [7]. By contrast, FEAR mutants merely show a delay in exit from mitosis [19]. Therefore, of these networks, only MEN activity is both necessary and sufficient for mitotic exit.

Unlike mitosis, the nuclear divisions of meiosis I and II must be achieved without triggering an intervening round of DNA replication. During meiosis I the spindle must elongate, dividing the nuclei, and be disassembled, a task under the control of Cdc14. However, DNA replication complexes must not be relicensed, a process also dependent on Cdc14 release [20]. Consequently, FEAR activation and CDK activity must be balanced. During exit from meiosis I, FEAR is activated, but MEN is not [21], [22]. The transient FEAR-based Cdc14 release must therefore be more effective during meiosis I than during mitosis to be able to disassemble the anaphase I spindle. Nevertheless, FEAR activation during exit from meiosis I must not fully inactivate CDK, which could lead to relicensing of replication origins.

Localisation changes [22] and modification [23] of the major meiotic cyclin Clb1 has been observed during meiosis I. In this report, we probed the functional significance of Clb1's nuclear localisation and its dependency on its phosphorylation. We have found that Clb1 localisation to the nucleus is required for its phosphorylation but not *vice versa*. We also provide evidence that increased nuclear localisation of Clb1 amplifies FEAR activation during meiosis. We suggest that the meiosis-specific nuclear localisation of Clb1 might facilitate FEAR-mediated disassembly of anaphase I spindles.

## Materials and Methods

The genotypes of all strains used in the experiments are listed in Table S1. Meiotic cultures were set up as previously described [24]. Preparation of whole cell extracts and *in situ* immunofluorescence were performed as previously described [24].

### Immunoprecipitation of Clb1

For meiotic samples, 25 ml samples of cell cultures at OD_600_ of 3 were used. For mitotic samples 75 ml samples of cell cultures at OD_600_ of 1 were used. Samples were bead beaten in lysis buffer (50 mM Hepes pH7.6, 75 mM KCl, 1 mM MgCl_2_, 1 mM EGTA, 0.1% NP40, 50 mM NaF, 60 mM β-glycerophosphate, 1 mM pefabloc, 1x Roche EDTA-free protease inhibitor cocktail) and cleared by spinning twice at 13 K rpm for 15 minutes at 4°C. The lysate was mixed with 1 µl of primary antibody (3F10: Rat-anti-HA Roche or 9E10: Mouse anti-myc Roche) and DTT (final concentration  = 1 mM) and incubated on a rotary wheel at 4°C for an hour. Pre-equilibrated Protein-G dynabeads were added to the lysate and incubated for further 2 hours. Beads were washed thrice in 1 ml lysis buffer. The washed beads were then used in phosphatase treatment or kinase assays.

### Phosphatase treatment of immunoprecipitated Clb1

50 µL of beads bearing bound Clb1 were washed and resuspended in 1x NEB buffer for Lambda phosphatase(with 2 mM MnCl_2_), and split into 3 samples. 1 µl of Lambda Protein Phosphatase (NEB) was added to the first sample. To the second sample, 1 µl of Lambda Protein Phosphatase (NEB) was added along with a mixture of phosphatase inhibitors (1 mM NaVnO_4_, 50 mM NaF, 5 mM EDTA and 0.1 µM Okadaic acid). The third sample served as a negative control. The three samples were then incubated at 30°C for 30 minutes with shaking. The reaction was stopped by adding 2x Laemmli sample buffer (8% SDS, 40% glycerol, 20% β-ME, 0.008% bromophenol blue and 0.125 M Tris-Cl pH6.8) and heating at 94°C for 5 minutes.

### Kinase Assay

35 µl of beads bearing bound protein were washed and resuspended in kinase assay buffer (50 mM Tris/HCL pH7.5, 10 mM MgCl_2_, 1 mM DTT and 5 mM β-glycerophosphate). The assay was performed at 30°C with 50 µM ATP (including 0.25 µCi/µl γ-32P-ATP) and 35 µM or 3.5 µM of Histone H1 for 40 minutes, with 25 µM 1-NM-PP1(+) or an equivalent volume of DMSO (-). Samples were taken at 0, 10, 20 and 40 minutes, and stopped by addition of Laemmli sample buffer and heating. Samples were run on 15% SDS-PAGE gels and analysed using an imaging plate and phosphorimager.

## Results

### Clb1 modification and nuclear localisation is specific to metaphase I of meiosis

**Figure 1:**
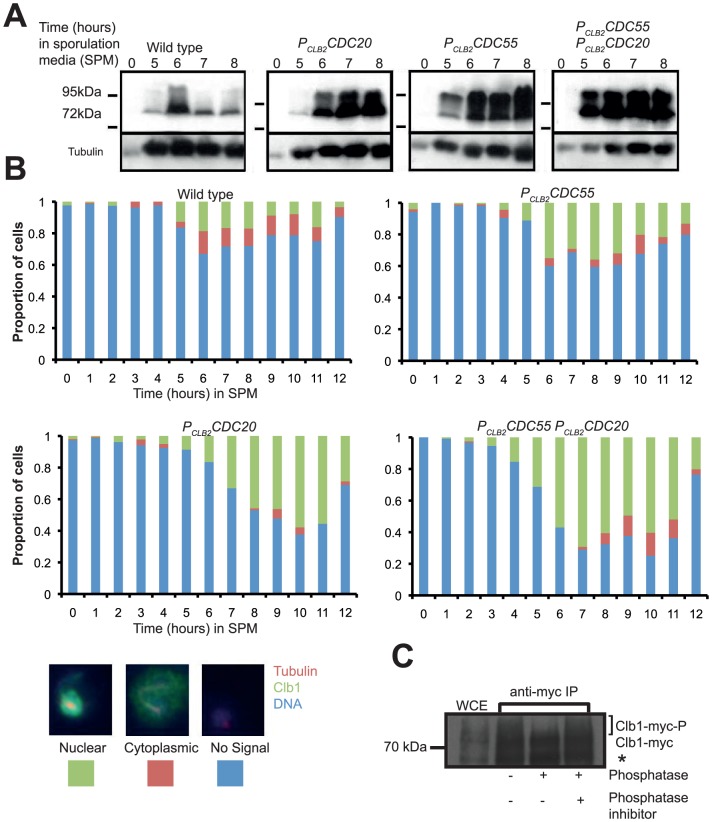
Clb1 is phosphorylated and localizes to the nucleus during metaphase I. Cultures of *CLB1-myc_9_*, *P_CLB2_CDC55 CLB1-myc_9_*, *P_CLB2_CDC20 CLB1-myc_9_* and *P_CLB2_CDC20 P_CLB2_CDC55 CLB1-myc_9_* strains were induced to enter meiosis by transferring them to SPM. A) Modification of Clb1 was assayed by subjecting whole cell extracts from the above cultures to SDS-PAGE followed by Western analysis using an anti-myc antibody. Tubulin served as a loading control. B) Cells were fixed and examined for Clb1-Myc localisation by *in situ* immunofluorescence. Proportion of cells with nuclear Clb1 (green), cytoplasmic Clb1 (red) and no Clb1 signals (blue) are indicated. A representative image of a cell belonging to each of the three categories is shown below the graphs. C) Clb1 was immunoprecipitated from a culture of *P_CLB2_CDC20 CLB1-myc_9_ cdc28-as* cells following 8 hours into SPM and then were either mock treated or incubated with λ-phosphatase alone or with λ-phosphatase + phosphatase inhibitors at 30°C for 30 minutes. Samples were analysed by SDS-PAGE followed by Western blotting. Asterisk indicates a Clb1 cleavage fragment.

Clb1 has been observed to be post-translationally modified and localized to the nucleus during meiosis I [22], [23]. We first tested whether progression through anaphase I was required for post-translational modification and nuclear localisation of Clb1 during meiosis. We constructed Clb1-tagged strains containing the *CDC20* (responsible for activating the anaphase promoting complex) either under the endogenous promoter or the mitosis-specific promoter *P_CLB2_* [25]. We induced cells to enter meiosis by transferring them to sporulation medium (SPM). While wild type cells underwent two rounds of nuclear division and formed 60% tetrads after 12 h in SPM, the *P_CLB2_CDC20* cells remained mononucleate during the entire experiment (Figure S1). After 6 h into SPM, Clb1 was modified and localized to the nucleus in both wild type and *P_CLB2_CDC20* cells (Figure 1A and 1B). This indicates that progression through anaphase I is not required for Clb1 modification and nuclear localization. However, in contrast to wild type cells, Clb1 modification and nuclear localization was sustained in *P_CLB2_CDC20* cells (Figure 1A and 1B). This suggests that passage through anaphase I reverses the modification and nuclear localization of Clb1. One possibility is that activation of FEAR during anaphase I might abolish nuclear localization and modification of Clb1. Consistent with this possibility, nuclear localization of Clb1 during meiosis is retained in FEAR mutants [22]. If FEAR was required for delocalization of Clb1, then its premature activation should prevent Clb1 nuclear localization. Since *P_CLB2_CDC55* cells release Cdc14 prematurely from the nucleolus but undergo other cell cycle events like securin degradation, cohesin cleavage and Clb3 expression (specific to meiosis II) normally, we monitored Clb1 nuclear localization and modification in *P_CLB2_CDC55* cells [24]. Clb1 nuclear localisation and modification was maintained in both *P_CLB2_CDC55 and P_CLB2_CDC55 P_CLB2_CDC20* strains (Figure 1A and 1B) suggesting that Clb1 nuclear localization and modification is not sensitive to premature FEAR activation [24].

To test whether Clb1 was phosphorylated during meiosis I, we purified Clb1 by immunoprecipitation from *P_CLB2_CDC20* cells after 8 h into SPM and incubated it either with lambda phosphatase or with lambda phosphatase and phosphatase inhibitors or without lambda protein phosphatase (Figure 1C). Phosphatase treatment caused the disappearance of the modified Clb1 band and addition of phosphatase inhibitors to the phosphatase prevented this disappearance, indicating that Clb1 is phosphorylated during meiosis I.

**Figure 2:**
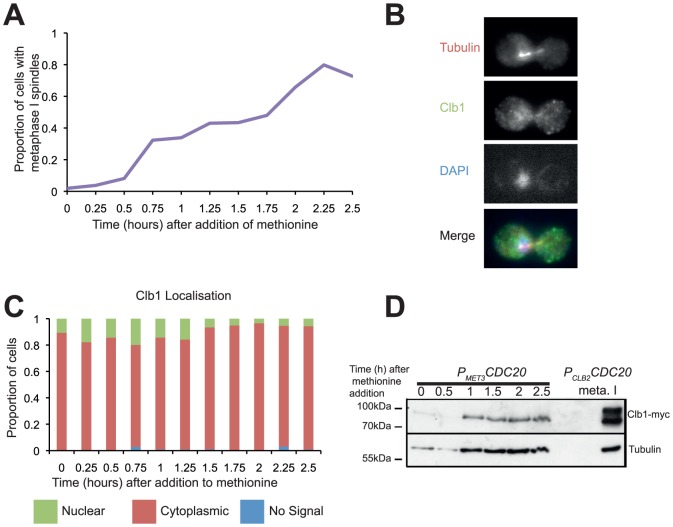
Clb1 modification and nuclear localisation are meiosis–specific. *P_MET3_CDC20 CLB1-myc_9_* cells were arrested in metaphase by transferring them to YEPD medium with 0.15% methionine for 2.5 hours. Culture samples were taken for *in situ* immunofluorescence and for preparing whole cell extracts. Localisation of Clb1-Myc and spindle formation was examined by *in situ* immunofluorescence. A) Percentage of cells containing metaphase spindles following addition of methionine is indicated. B) Representative image of a cell arrested in metaphase displaying Clb1 localisation is shown. C) Localization of Clb1 during the experiment is graphically presented (green-nuclear, red-cytoplasmic, blue-no signal). D) Whole cell extracts were subjected to SDS-PAGE followed by Western analysis for assaying Clb1 phosphorylation. For comparison, whole cell extract from diploid *P_CLB2_CDC20 CLB1-myc_9_* cells after 8 hours in SPM was included in the gel.

We then tested whether Clb1 modification and nuclear localisation also occur during a mitotic metaphase arrest. *P_MET3_CDC20* cells expressing a TAP tagged version of Clb1 were arrested in metaphase by Cdc20 depletion, 80% of cells had metaphase spindles 2.5 hours after resuspension in medium containing methionine, indicating that a majority of cells were arrested in metaphase (Figure 2A). However Clb1 was neither modified nor enriched in the nucleus (Figure 2B–D). Thus, phosphorylation and nuclear localization of Clb1 are meiosis-specific.

### CDK and Cdc5 activity are required for Clb1 phosphorylation

The meiosis-specific nature of Clb1 phosphorylation led us to consider Ime2 as a candidate kinase. Ime2 is a meiosis specific kinase related to CDK with key roles in the control of meiosis [26]. Moreover, there are five Ime2 consensus sites in Clb1, including a complete consensus site [27] in proximity to the nuclear localisation sequence. Using an analogue-sensitive allele of *IME2, (ime2-as)*, we examined whether Ime2 is required for nuclear localization and phosphorylation of Clb1-TAP. Addition of the inhibitor, 1-NA-PP1, selectively inhibits Ime2-as but not Ime2 [28] [Figure S2]. We performed this experiment in *P_CLB2_CDC20* strain background to sustain Clb1 modification and nuclear localization. Inhibition of Ime2 by addition of the inhibitor to the cultures after 6, 7, or 8 hours into SPM did not reduce the nuclear concentration or phosphorylation of Clb1-TAP (Figure S2). Addition of the inhibitor at 5 hours led to extremely reduced Clb1 expression (Figure S2) which is consistent with Ime2's role in exit from pachytene [29].

**Figure 3:**
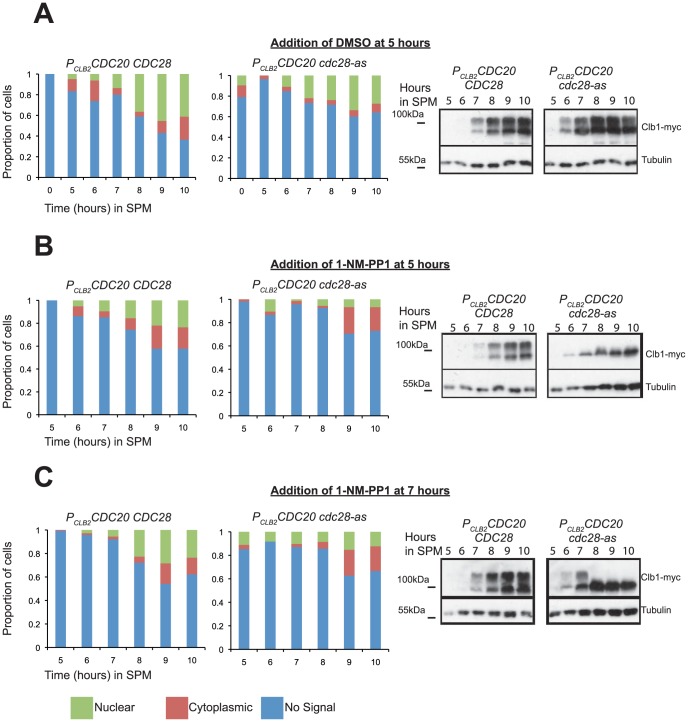
CDK activity is required for Clb1 phosphorylation and nuclear localization during meiosis I. Cultures of *CDC28 P_CLB2_CDC20 CLB1-myc_9_* cells and *cdc28-as P_CLB2_CDC20 CLB1-myc_9_* cells were induced to enter meiosis by transferring them to SPM. 1-NM-PP1 (10 µM) was added to the cultures after 5, 6, 7 and 8 hours in SPM. As a control, an equivalent amount of DMSO was added to the cultures after 5 h in SPM. Samples were taken hourly from 5 hours onwards for whole cell extracts and for *in situ* immunofluorescence. Whole cell extracts were analysed by Western blotting using anti-myc and anti-tubulin antibodies. Localization of Clb1 was assayed by *in situ* immunofluorescence (green-nuclear, red-cytoplasmic, blue-no signal). Western blotting and Clb1 localization data for the three cultures DMSO/5 h, 1-NM-PP1/5 h and 1-NM-PP1/7 h are indicated in A, B and C respectively. Data for additional time points are indicated in the Figure S3.

We then tested whether CDK activity is required for Clb1 phosphorylation and nuclear localization using an analogue-sensitive allele *cdc28-as* [28]. Addition of the inhibitor at 5 hours completely prevented both the modification and nuclear localisation of Clb1 (Figure 3A and 3B). Addition of the ATP analogue 1-NM-PP1 at either 6, 7 or 8 hours after transfer to SPM abolished Clb1 modification and reduced its nuclear localisation (Figure 3C and Figure S3). These results indicate that Clb1 modification and nuclear localisation are dependent on CDK but not Ime2 activity.

### Cdc5 activity is required for Clb1 modification but not nuclear localization

**Figure 4:**
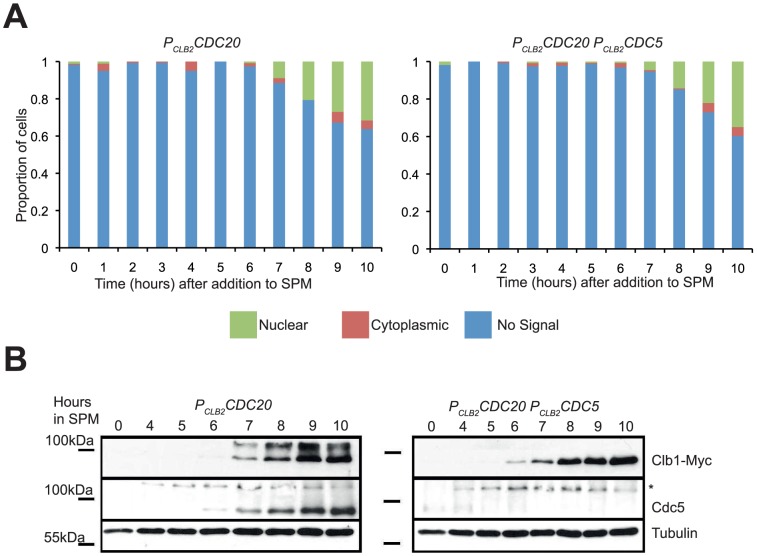
Cdc5 is required for phosphorylation, but not nuclear localisation, of Clb1 during meiosis I. *P_CLB2_CDC20 CLB1-myc_9_* and *P_CLB2_CDC5 P_CLB2_CDC20 CLB1-myc_9_* cells were induced to enter meiosis by transferring them to SPM. A) Samples were taken hourly throughout the time course for *in situ* immunofluorescence to determine Clb1 localisation (green-nuclear, red-cytoplasmic, blue-no signal). B) Samples were taken hourly from 4 hours for preparing whole cell extracts. Whole cell extracts were analysed by Western blotting using anti-myc (Clb1), anti-Cdc5 and anti-tubulin antibodies. Asterisk represents a cross-reactive band seen in anti-Cdc5 blots.

CDK is not meiosis-specific; to explain the meiosis-specific nature of the phosphorylation, another regulatory step must be involved. Cdc5 is also active during meiosis and has a number of meiosis-specific functions [30], [31]. Cdc5 expression also coincides with Clb1 phosphorylation (Figure S3). The requirement for Cdc5 activity was examined by depleting Cdc5 in meiosis by replacing the endogenous promoter with that of *CLB2*. *P_CLB2_CDC5* cells undergoing meiosis expressed Clb1, but it was not phosphorylated (Figure 4A), indicating that Cdc5 is required for phosphorylation of Clb1. However, Clb1 was still concentrated in the nucleus in *P_CLB2_CDC5* cells (Figure 4B), indicating that Cdc5 activity is not required for nuclear localisation of Clb1. This also shows that Clb1 nuclear localisation is independent of its phosphorylation. Since Cdc5 is required for exit from meiotic prophase [31], our results also indicate that exit from prophase is not required for nuclear localisation of Clb1.

### Functional significance of Clb1 nuclear localisation during meiosis I

**Figure 5:**
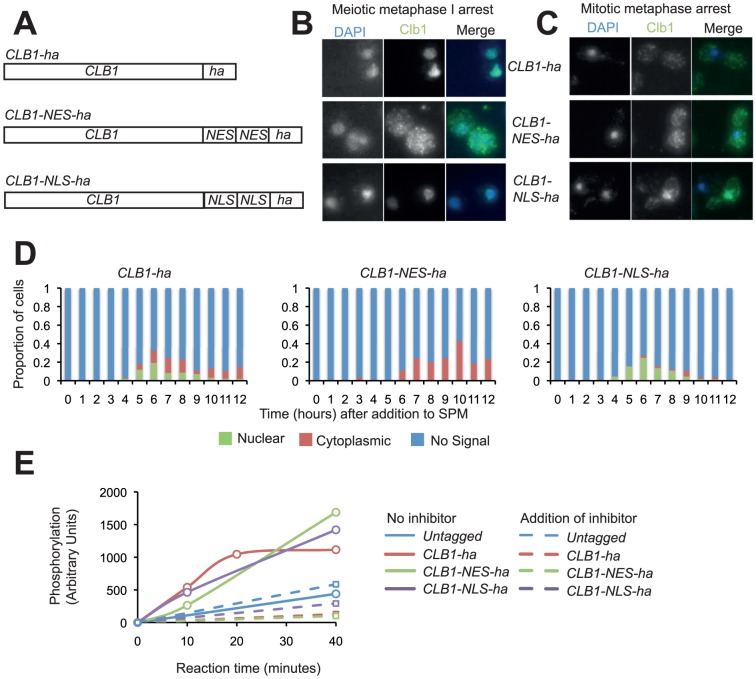
Fusion of NES/NLS sequences to Clb1 alters its nuclear localization without affecting its kinase activity. A) Schematic showing the variants of Clb1 constructed to alter its nuclear localization. Tandem copies of Nuclear Localization sequences (NLS) or Nuclear Export Sequences (NES) followed by a single copy of HA epitope was added the C-terminus of Clb1. B) Diploid cells bearing either wild type Clb1 or NES/NLS –tagged Clb1 and containing *CDC20* under the *CLB2* promoter were arrested in metaphase I by transferring them to SPM for 7 h. Clb1 localization was determined by immunofluorescence. Note that Clb1 is diffuse in the NES-tagged strain but nuclear in wild type/NLS-tagged strains. C) Cells bearing either wild type Clb1 or NES/NLS –tagged Clb1 and containing *CDC20* under the repressible *MET3* promoter were arrested in metaphase by Cdc20 depletion and Clb1localization was determined by immunofluorescence Notice that Clb1 is nuclear in the NLS-tagged strain but delocalized in wild type and NES-tagged strains (images of cells after 2 hours in methionine are shown). D) *CLB1-ha PDS1-myc_18_ cdc28-as*, *CLB1-NES-ha PDS1-myc_18_ cdc28-as* and *CLB1-NLS-ha PDS1-myc_18_ cdc28-as* cells were induced to enter meiosis by transferring them to SPM. Samples were taken hourly to determine Clb1 localisation. E) Tagged Clb1 was immunoprecipitated from mitotic cultures of *P_CLB2_CDC20 cdc28-as*, *CLB1-ha P_CLB2_CDC20 cdc28-as*, *CLB1-NES-ha P_CLB2_CDC20 cdc28-as*, and *CLB1-NLS-ha P_CLB2_CDC20 cdc28-as* cells (Figure S4). Purified Clb1 was incubated with 35 µM histone and 50 µM ATP (0.25 µCi/µL of γ-P^32^-ATP) in kinase buffer for 40 minutes, with samples taken at 0, 10, 20 and 40 minutes in the presence or absence of the inhibitor 1-NM-PP1. Samples were analysed by SDS-PAGE and gels were dried, exposed to phosphorimager screen and the signals were quantified using IMAGE Quant.

To determine the functional significance of Clb1 nuclear localisation, the protein was tagged with ectopic Nuclear Localisation Sequences (NLS) or Nuclear Export Sequences (NES), and 6 copies of HA epitope. In each case, two sequences were added in tandem to overcome any endogenous localisation signals (Figure 5A). The tandem NLS tag leads to nuclear localisation of Clb1 during mitosis (Figure 5C). In contrast the tandem NES severely reduced the nuclear localisation of Clb1 during meiosis I (Figure 5B, D). Crucially, the fusion of NLS/NES had little or no effect on Clb1-CDK activity (Figure 5E, Figure S4B). The *clb1*Δ strains sporulate poorly, producing only 15% tetrads in comparison to 70% for wild-type cells (Figure S4C). Expression of NES/NLS-tagged Clb1 does not affect sporulation efficiency in otherwise wild-type cells (Figure S4C), suggesting that the localization mutants are not inactivated, and that nuclear localisation of Clb1 is not essential for meiotic nuclear divisions. Spore viability was also largely unaffected by the mutations: *CLB1-ha* and *CLB1-NES-ha* strains have viabilities of 97%, whereas the *CLB1-NLS-ha* strain has a spore viability of 85%.

**Figure 6:**
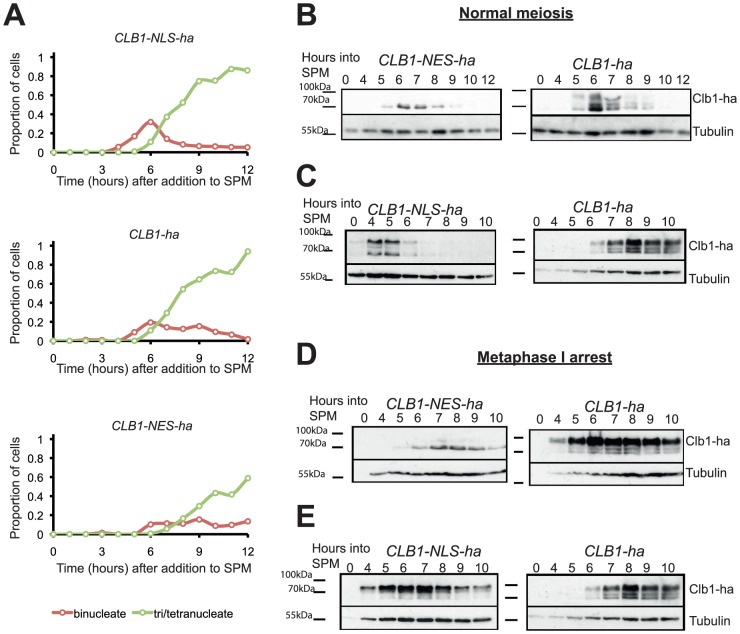
Phosphorylation of Clb1 during meiosis requires its localization to the nucleus. A) Cultures of *CLB1-ha PDS1-myc_18_, CLB1-NES-ha PDS1-myc_18_* and *CLB1-NLS-ha PDS1-myc_18_* cells were induced to enter meiosis by transferring them to SPM. Nuclear division in the three cultures was determined by DAPI staining and the data are represented graphically. B) Cultures of *CLB1-ha PDS1-myc_18_* and *CLB1-NES-ha PDS1-myc_18_* cells were induced to enter meiosis by transferring them to SPM. Whole cell extracts were analysed by Western blotting for Clb1 phosphorylation. C) Cultures of *CLB1-ha PDS1-myc_18_* and *CLB1-NLS-ha PDS1-myc_18_* cells were induced to enter meiosis by transferring them to SPM. Whole cell extracts were analysed by Western blotting for Clb1 phosphorylation. D) Cultures of *P_CLB2_CDC20 CLB1-ha* and *P_CLB2_CDC20 CLB1-NES-ha* cells were induced to enter meiosis by transferring them to SPM. Samples were taken at time 0 and hourly from 4–10 hours for whole cell extracts and analysed by Western blotting. E) Cultures of *P_CLB2_CDC20 CLB1-ha* and *P_CLB2_CDC20 CLB1-NLS-ha* cells were induced to enter meiosis by transfer to SPM. Samples were taken at time 0 and hourly from 4–10 hours for whole cell extracts and analysed by Western blotting.

To determine the effect of altering nuclear localization of cyclin Clb1 during meiosis, we induced wild type and nuclear localization mutant strains to enter meiosis and examined nuclear division and Clb1 phosphorylation (Figure 6A–C). We found that the Clb1-NES-ha was not phosphorylated unlike wild type Clb1 and Clb1-NLS-ha. To confirm this, we assayed modification of wild type/NLS-/NES versions of Clb1 in *P_CLB2_CDC20* strains, in which Clb1 phosphorylation is maintained (Figure 6D, E). The Clb1-NES-ha was largely unmodified after 7 hours into SPM. In contrast, Clb1-NLS-ha was heavily phosphorylated in comparison to wild type Clb1. These results concur with the idea that nuclear import of Clb1 is a prerequisite for its modification during meiosis I. We therefore propose that Clb1 is imported to the nucleus by a meiosis-specific mechanism, and phosphorylated in a Cdc5-dependent manner upon nuclear entry.

### Clb1 localisation and FEAR regulation

**Figure 7:**
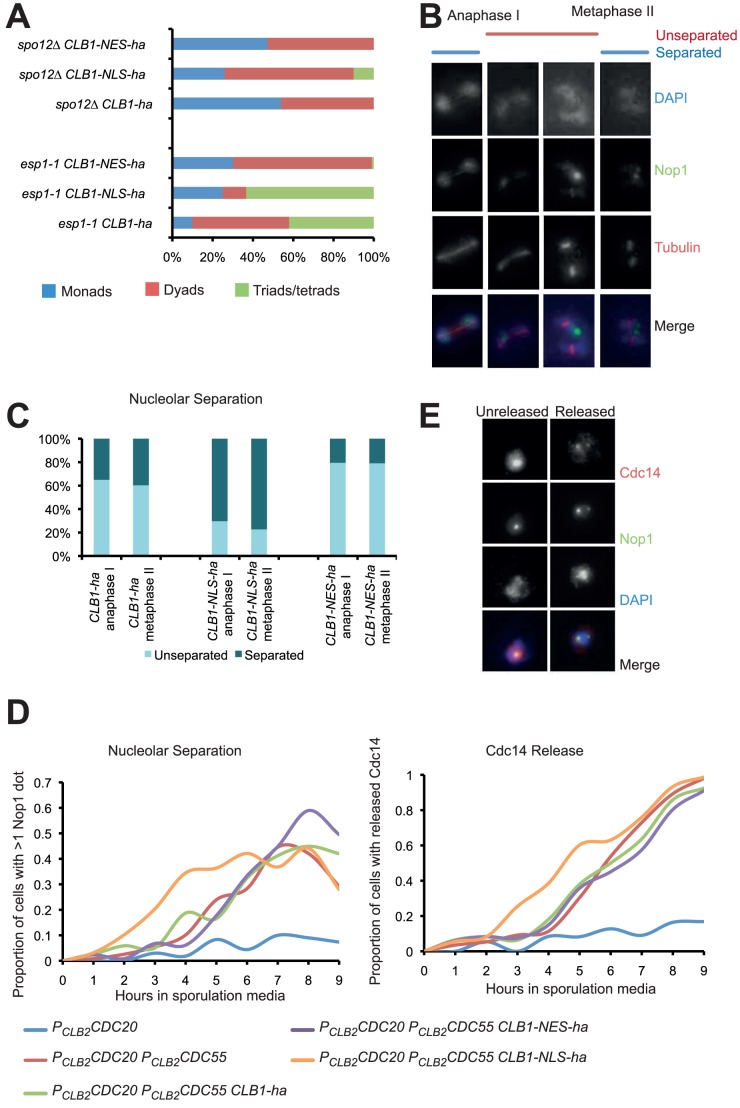
Increased nuclear localization of Clb1 promotes FEAR activation during meiosis. A) Diploid *CLB1, CLB1-NES* and *CLB1-NLS* strains bearing *spo12Δ* and *esp1-1* or their wild type alleles were allowed to sporulate. Percentage of cells forming dyads, tetrads and monads were calculated after 48 h. B) Cultures of *CLB1-ha PDS1-myc_18_*, *CLB1-NES-ha PDS1-myc_18_*, and *CLB1-NLS-ha PDS1-myc_18_* cells were induced to enter meiosis by transferring them to SPM. Nucleolar separation in cells containing anaphase I and metaphase II spindles was assayed by immunofluorescence. Representative images of cells are shown on the right. C) Data obtained in B are presented graphically. D) Nucleolar separation and nucleolar release of Cdc14 in the sporulating cultures of *P_CLB2_CDC20* (blue), *P_CLB2_CDC20 P_CLB2_CDC55* (red), *P_CLB2_CDC20 P_CLB2_CDC55 CLB1-ha* (green), *P_CLB2_CDC20 P_CLB2_CDC55 CLB1-NES-ha* (purple) and *P_CLB2_CDC20 P_CLB2_CDC55 CLB1-NLS-ha* (orange) strains were assayed by immunofluorescence and the data are graphically presented. E) Sample images of cells with nucleolar Cdc14 or Cdc14 released are shown on the right.

Activation of FEAR during meiosis I but not during mitosis is sufficient to cause disassembly of anaphase spindles. We therefore tested whether nuclear localisation of Clb1 amplifies FEAR during meiosis. We first tested whether FEAR mutants show a genetic interaction with Clb1 localisation mutants (Figure 7A). *spo12*Δ and *esp1-2* mutant cells do not activate FEAR efficiently during meiosis I and fail to disassemble anaphase I spindles [22]. The mutant cells undergo two divisions on the same spindle forming dyads. *CLB1-NLS-ha* but not *CLB1-NES-ha* partially suppressed the dyad phenotype of both *spo12*Δ and *esp1-2* mutant strains (Figure 7A). This is consistent with the possibility that increased nuclear localization of Clb1 promotes FEAR activation during meiosis I.

To determine whether increased nuclear localization of Clb1 promotes efficient activation of FEAR, we compared the kinetics of nucleolar separation in wild type and nuclear localization mutants of *CLB1*. Separation of rDNA is triggered by the release of Cdc14 [32], [33] and is an indirect measure of FEAR activation. We quantified nucleolar splitting in cells with either anaphase I or metaphase II spindles. Interestingly, the fraction of *CLB1-NLS-ha* cells with separated nucleoli during anaphase I and metaphase II spindles was 1.5 -2 fold greater than in wild type cells (Figure 7B, C). In contrast, a lesser proportion of *CLB1-NES-ha* cells had separated nucleoli in comparison to wild type cells. This is consistent with the idea that nuclear localization of Clb1 increases the efficiency of FEAR activation.

Cdc14 is released prematurely from the nucleolus in *P_CLB2_CDC55 P_CLB2_CDC20* strains [24], [34] as PP2A^Cdc55^ is not present to counter Net1 phosphorylation by CDK. To test whether increased nuclear localization of Clb1 amplifies FEAR activation, we measured the kinetics of Cdc14 release and nucleolar division in *P_CLB2_CDC55 P_CLB2_CDC20* strains in wild type and nuclear localisation mutants of *CLB1* through meiosis. Cells expressing NLS-tagged Clb1 released Cdc14 and separated nucleoli earlier in comparison to wild type cells (Figure 7D and 7E). This supports the idea that the meiosis-specific nuclear localization of Clb1 amplifies FEAR activation. However the NES-tagged Clb1 had little effect on the kinetics of Cdc14 release and nucleolar division. It is possible that other cyclins might compensate for the decreased nuclear levels of cyclins in *CLB1-NES-ha* cells. Moreover the NES tag does not fully deplete Clb1 from the nucleus (Figure 5). Taken together our results are consistent with the hypothesis that increased nuclear localization of Clb1 enhances activation of FEAR during meiosis I.

## Discussion

Phosphorylation and nuclear localization of Clb1 during meiosis in budding yeast has been previously reported [22], [23]. However the functional significance of these observations and the mechanistic correlation between the two events, if any, was not known. In this paper, we demonstrate that phosphorylation and nuclear localization of Clb1 do not occur during mitosis and are therefore meiosis-specific. Phosphorylation of Clb1 is dependent on Cdc5 and CDK activity. In contrast, Clb1 nuclear localisation depends on CDK but not on Cdc5 activity. Phosphorylation of Clb1 requires its localization to the nucleus but not *vice versa*. Since Clb1 is the major cyclin for meiosis I and Clb-CDK driven FEAR is responsible for exit from meiosis I, we tested whether the meiosis-specific behaviour of Clb1 affects FEAR activation. We found that increasing nuclear localisation of Clb1 partially rescued the nuclear division defect of FEAR mutants, and advanced the timing of FEAR activation during meiosis I. Altering Clb1 localisation during meiosis did not lead to a loss of sporulation efficiency or viability in otherwise wild-type backgrounds, implying that Clb1 localisation is not essential for successful meiosis. Functional redundancy amongst B-cyclins could mask the effects of altering Clb1 nuclear localization. However the synthetic effects observed with FEAR mutants suggests that Clb1 nuclear localization contributes to efficient exit from meiosis I. Our data support the idea that Clb1 localisation to the nucleus might amplify FEAR activation during exit from meiosis I and cause disassembly of anaphase I spindles.

Increased nuclear localization of Clb1 could result in enhanced access to its nucleolar substrate Net1. Moreover Clb1's nuclear localization is unaffected by premature activation of Cdc14 (Figure 1B) and therefore phosphorylation of Net1 could proceed at an increased rate (compared to mitotic anaphase) despite FEAR activation. Alternatively, nuclear Clb1 could indirectly amplify FEAR activation. Mutations in genes encoding two karyopherins Mtr104 and Srp1 suppress the null phenotype of MEN mutants[35]. It will be interesting to test whether these karyopherins regulate nuclear localization of Clb1 during meiosis I.

Our work raises a number of interesting questions. How Clb1 is transported to the nucleus during meiosis I? Does Cdc5 directly phosphorylate Clb1 or indirectly, by promoting degradation of CDK inhibitor Swe1? What is the functional significance of Clb1 phosphorylation? How is Clb1 phosphorylation and nuclear localization reversed after meiosis I? Answers to these questions might help in understanding how meiotic yeast cells modulate their CDK activity to undergo two rounds of nuclear division without an intervening S-phase.

## Supporting Information

Figure S1 **Nuclear division in *CLB1-myc_9_* cells.** Cultures of *CLB1-myc_9_* strains were induced to enter meiosis by transferring them to SPM. Samples were taken for *in situ* immunofluorescence hourly and nuclear division was scored by DAPI staining.
(EPS)

Figure S2 **Kinase activity of Ime2 is not required for nuclear localization and phosphorylation of Clb1.** Cultures of *IME2 P_CLB2_-CDC20 CLB1-TAP* cells and *ime2-as P_CLB2_CDC20 CLB1-TAP* cells were induced to enter meiosis by transferring them to SPM. The ATP analogue 1-NA-PP1 (20 µM) or an equivalent volume of DMSO was added to sporulating cultures at 5, 6, 7 and 8 hours into SPM. A) Modification of Clb1 was assayed by subjecting whole cell extracts from the above cultures to SDS-PAGE followed by Western analysis using an anti-TAP antibody. Blots were also probed with anti-myc (to detect Ime2) and anti-tubulin antibody. B) Cells were fixed and examined for Clb1-Myc localisation by *in situ* immunofluorescence. Proportion of cells with nuclear Clb1 (green), cytoplasmic Clb1 (red) and no Clb1 signals (blue) are indicated.
(EPS)

Figure S3 **CDK activity is required for Clb1 phosphorylation and nuclear localization.** Cultures of *CDC28 P_CLB2_-CDC20 CLB1-myc_9_* cells and *cdc28-as P_CLB2_CDC20 CLB1-myc_9_* strains were induced to enter meiosis by transferring them to SPM. Samples were taken hourly from 5 hours for whole cell extracts (A) and hourly throughout the time course for imaging (B). 10 µM 1-NM-PP1 was added to sporulating cultures at 6 and 8 hours into SPM.
(EPS)

Figure S4 **Fusion of NES and NLS sequences to the C-terminus of Clb1 does not affect CDK-Clb1 activity.** Tagged Clb1 was immunoprecipitated from mitotic cultures of *P_CLB2_CDC20 cdc28-as*, *CLB1-ha P _CLB2_CDC20 cdc28-as*, *CLB1-NES-ha P_CLB2_CDC20 cdc28-as* and *CLB1-NLS-ha P_CLB2_CDC20 cdc28-as* strains using an anti-HA antibody. A) Immunoprecipitated Clb1 from the three cultures was detected by western blotting. B) Purified Clb1 was incubated with 3.5 µM histone and 50 µM ATP (0.25 µCi/µL of γ-32-ATP) in the kinase buffer for 40 minutes and phosphorylation of Histone H1 after 0, 10, 20 and 40 minutes (in the absence of the inhibitor 1-NM-PP1) was determined. C) Sporulation efficiencies of diploid strains bearing Clb1 Nuclear Localization sequence or Nuclear Export sequences with or without *clb1Δ* or *clb3Δclb4Δ* are graphically presented.
(EPS)

Table S1 **List of yeast strains.** All yeast strains used are derivatives of SK1 and have the following markers, unless otherwise stated stated. *ho::LYS2/ho::LYS2, ura3/ura3, leu2::hisG/leu2::hisG, trp1::hisG/trp1::hisG, his3::hisG/his3::hisG, lys2/lys2. The* strains used for experiments described in the Main and Supplementary figures are listed in Table 1A and 1B respectively.
(DOCX)
