# Correction: CDK-Dependent Nuclear Localization of B-Cyclin Clb1 Promotes FEAR Activation during Meiosis I in Budding Yeast

**DOI:** 10.1371/journal.pone.0099688

**Published:** 2014-06-02

**Authors:** The PLOS ONE Staff

Figure 6 is incorrect. Panels 6C and 6E in Figure 6 are duplicated. Panel 6E is incorrectly displayed as panel 6C. The authors apologize for this error and have provided a revised Figure 6 with the correct panel 6C, which can be viewed here. The raw blots for Figure 6 are also available with this correction.

**Figure 6:**
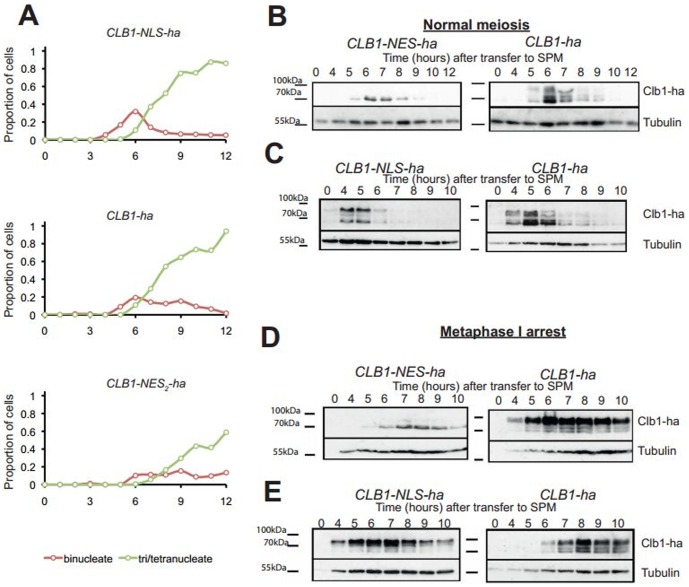
Phosphorylation of Clb1 during meiosis requires its localization to the nucleus. A) Cultures of *CLB1-ha PDS1-myc_18_, CLB1-NES-ha PDS1-myc_18_* and *CLB1-NLS-ha PDS1-myc_18_* cells were induced to enter meiosis by transferring them to SPM. Nuclear division in the three cultures was determined by DAPI staining and the data are represented graphically. B) Cultures of *CLB1-ha PDS1-myc_18_* and *CLB1-NES-ha PDS1-myc_18_* cells were induced to enter meiosis by transferring them to SPM. Whole cell extracts were analysed by Western blotting for Clb1 phosphorylation. C) Cultures of *CLB1-ha PDS1-myc_18_* and *CLB1-NLS-ha PDS1-myc_18_* cells were induced to enter meiosis by transferring them to SPM. Whole cell extracts were analysed by Western blotting for Clb1 phosphorylation. D) Cultures of *P_CLB2_CDC20 CLB1-ha* and *P_CLB2_CDC20 CLB1-NES-ha* cells were induced to enter meiosis by transferring them to SPM. Samples were taken at time 0 and hourly from 4–10 hours for whole cell extracts and analysed by Western blotting. E) Cultures of *P_CLB2_CDC20 CLB1-ha* and *P_CLB2_CDC20 CLB1-NLS-ha* cells were induced to enter meiosis by transfer to SPM. Samples were taken at time 0 and hourly from 4–10 hours for whole cell extracts and analysed by Western blotting.

## Supporting Information

Figure S1 **Raw blot 6B loading control**
(TIF)

Figure S2 **Raw blot 6C loading control**
(TIF)

Figure S3 **Raw blot 6D loading control**
(TIF)

Figure S4 **Raw blot 6E loading control**
(TIF)

Figure S5 **Raw blot 6B**
(TIF)

Figure S6 **Raw blot 6C**
(TIF)

Figure S7 **Raw blot 6D**
(TIF)

Figure S8 **Raw blot 6E**
(TIF)
