# Supplementary material for: CDK-Dependent Nuclear Localization of B-Cyclin Clb1 Promotes FEAR Activation during Meiosis I in Budding Yeast
Source: PLoS One. 2013 Nov 1;8(11):e79001. doi: 10.1371/journal.pone.0079001 (PMC3815228; doi:10.1371/journal.pone.0079001)
Supplement: Table S1 — List of yeast strains. All yeast strains used are derivatives of SK1 and have the following markers, unless otherwise stated stated. ho::LYS2/ho::LYS2, ura3/ura3, leu2::hisG/leu2::hisG, trp1::hisG/trp1::hisG, his3::hisG/his3::hisG, lys2/lys2. The strains used for experiments described in the Main and Supplementary figures are listed in Table 1A and 1B respectively. (DOCX) [file pone.0079001.s005.docx]

**Supporting Information Table S1**

**Table S1A** (strains used for experiments in the Main figures)

| Strain Number | Genotype | Used in figure |
| --- | --- | --- |
| 2311 | SK1 MATa/ α *cdc20::P_CLB2_CDC20::kanMX6* *CLB1-myc9::klTRP1* | 1,4 |
| 2312 | SK1 MATa/α *cdc20::P_CLB2_CDC20::kanMX6*  *CLB1-myc9::klTRP1* *cdc55:P_CLB2_CDC55:KanMX6* | 1 |
| 2348 | SK1 MATa/α *CLB1-myc9::klTRP1* *cdc55:P_CLB2_CDC55:KanMX6* | 1 |
| 2350 | SK1 MATa/α *CLB1-myc9::klTRP1* | 1 |
| 2378 | SK1 MATa *CLB1-myc9::klTRP1 cdc20::P_MET3_CDC20::TRP1* | 2 |
| 2597 | SK1 MATa/α *cdc20::P_CLB2_CDC20::kanMX6* *CLB1-myc9::klTRP1* | 3 |
| 2600 | SK1 MATa/α *cdc28-as1* *CLB1-myc_9_::klTRP1 cdc20::P_CLB2_CDC20::kanMX6* | 1,3,5 |
| 2893 | SK1 MATa/α *CLB1-myc9::klTRP1 P_CLB2_CDC5::HIS3MX6 cdc20::P_CLB2_CDC20::kanMX6* | 4 |
| 1738 | SK1 MATa/ α | 5 |
| 2567 | SK1 MATa/α *clb3::natMX4 clb4::hphMX4* | 5 |
| 2615 | SK1 MATa/α *clb1Δ:HIS3MX6* | 5 |
| 3078 | SK1 MATa/α *cdc28-as1CLB1-NLS,NLS-ha::HIS3MX6 cdc20::P_CLB2_CDC20::KanMX6* | 5 |
| 3073 | SK1 MATa/α *cdc28-as1 CLB1-ha_6_:HIS3MX6 cdc20::P_CLB2_CDC20::kanMX6* | 5 |
| 3081 | SK1 MATa/α *cdc28-as1* *CLB1-NES,NES-ha::HIS3MX6* *cdc20::P_CLB2_CDC20::KanMX6* | 5 |
| 2858 | SK1 MATa/α *PDS1-myc18::TRP1*(K.lactis) *CLB1-NLS,NLS-ha::HIS3MX6* | 5,6,7 |
| 2542 | SK1 MATa/α *PDS1-myc_18_ ::TRP1(K. lactis) CLB1-ha_6_:HIS3MX6* | 5,6,7 |
| 2550 | SK1 MATa/α *PDS1-myc_18_ ::TRP1(K.lactis) CLB1-NES,NES-ha::HIS3MX6* | 5,6,7 |
| 2881 | SK1 MATa/α *PDS1-myc_18_::TRP1(K.lactis) CLB1-NLS,NLS-ha::HIS3MX6 cdc20::P_CLB2_CDC20::KanMX6* | 5,6 |
| 2674 | SK1 MATa/α *CLB1-ha_6_::HIS3MX6 cdc20::P_CLB2_CDC20::KanMX6* | 5,6 |
| 2640 | SK1 MATa/α *cdc20::P_CLB2_CDC20::KanMX6 CLB1-NES,NES-ha::HIS3MX6* | 5,6 |
| 2952 | SK1 MAT a/α *spo12Δ ::KanMX6 CLB1-NLS,NLS-ha::HIS3MX6* | 7 |
| 2946 | SK1 MAT α/a *spo12Δ ::KanMX6 CLB1-ha_6_:HIS3MX6* | 7 |
| 2949 | SK1 MAT a/α *spo12Δ::KanMX6 CLB1-NES,NES-ha::HIS3MX6* | 7 |
| 3007 | SK1 MATa/α *esp1-2 CLB1-NLS-ha:HIS3MX6* | 7 |
| 3006 | SK1 MATa/α *esp1-2 CLB1-ha_6_:HIS3MX6* | 7 |
| 3008 | SK1 MATα/a *esp1-2 CLB1-NES,NES-ha::HIS3* | 7 |
| 2119 | SK1 MATa/α *net1Δ::HIS3MX6 trp1::NET1-TEV-myc_9_::TRP1 cdc20:P_CLB2_CDC20-KanMX6* | 7 |
| 2120 | SK1MATa/α *net1Δ::HIS3MX6 trp1::NET1-TEV-myc_9_::TRP1 cdc55:P_CLB2_CDC55-KanMX6 cdc20:P_CLB2_CDC20-KanMX6* | 7 |
| 3057 | SK1 MATa/α *net1Δ::HIS3MX6 trp1::NET1-TEV-myc9::TRP1 cdc55:P_CLB2_CDC55-KanMX6 cdc20:P_CLB2_CDC20-KanMX6 CLB1-NLS,NLS-ha_6_:HIS3* | 7 |
| 3015 | SK1 MATa/α *net1Δ::HIS3MX6 trp1::NET1-TEV-myc9::TRP1 cdc55:P_CLB2_CDC55-KanMX6 cdc20:P_CLB2_CDC20-KanMX6 CLB1-ha_6_:HIS3* | 7 |
| 3016 | SK1 MATa/α *net1Δ::HIS3MX6 trp1::NET1-TEV-myc_9_::TRP1 cdc55:P_CLB2_CDC55-KanMX6 cdc20:P_CLB2_CDC20-KanMX6 CLB1-NES,NES-ha_6_:HIS3MX6* | 7 |

**Table S1B** (strains used for experiments in the Supporting Information figures)

| 2350 | SK1 MATa/ α *CLB1-myc_9_ :: klTRP1* | S1 |
| --- | --- | --- |
| 2452 | SK1 MATa/α *CLB1-TAP::TRP1 cdc20::P_CLB2_CDC20::KanMX6 ime2: ime2-as1 myc::TRP1(M146G)* | S2 |
| 2366 | SK1 MATa/α *CLB1-TAP::TRP1 cdc20::P_CLB2_CDC20::KanMX6* | S2 |
| 2597 | SK1 MATa/α *CLB1-myc9::klTRP1 cdc20::P_CLB2_ CDC20::kanMX6* | S3 |
| 2600 | SK1 MATa/α *cdc28-as1 CLB1-myc_9_::klTRP1 cdc20::P_CLB2_CDC20::KanMX6* | S3,S4 |
| 3078 | SK1 MATa/α *cdc28-as1 CLB1-NLS,NLS-ha::HIS3MX6 cdc20::P_CLB2_CDC20::kanMX6* | S4 |
| 3073 | SK1 MATa/α *cdc28-as1 CLB1-ha_6_:HIS3MX6 cdc20::P_CLB2_CDC20::KanMX6* | S4 |
| 3081 | SK1 MATa/α *cdc28-as1 CLB1-NES,NES-ha::HIS3MX6 cdc20::P_CLB2_CDC20::KanMX6* | S4 |
