# Supplementary figures and images for: Correction: CDK-Dependent Nuclear Localization of B-Cyclin Clb1 Promotes FEAR Activation during Meiosis I in Budding Yeast
Source: PLoS One. 2014 Jun 2;9(6):e99688. doi: 10.1371/journal.pone.0099688 (PMC4041849; doi:10.1371/journal.pone.0099688)

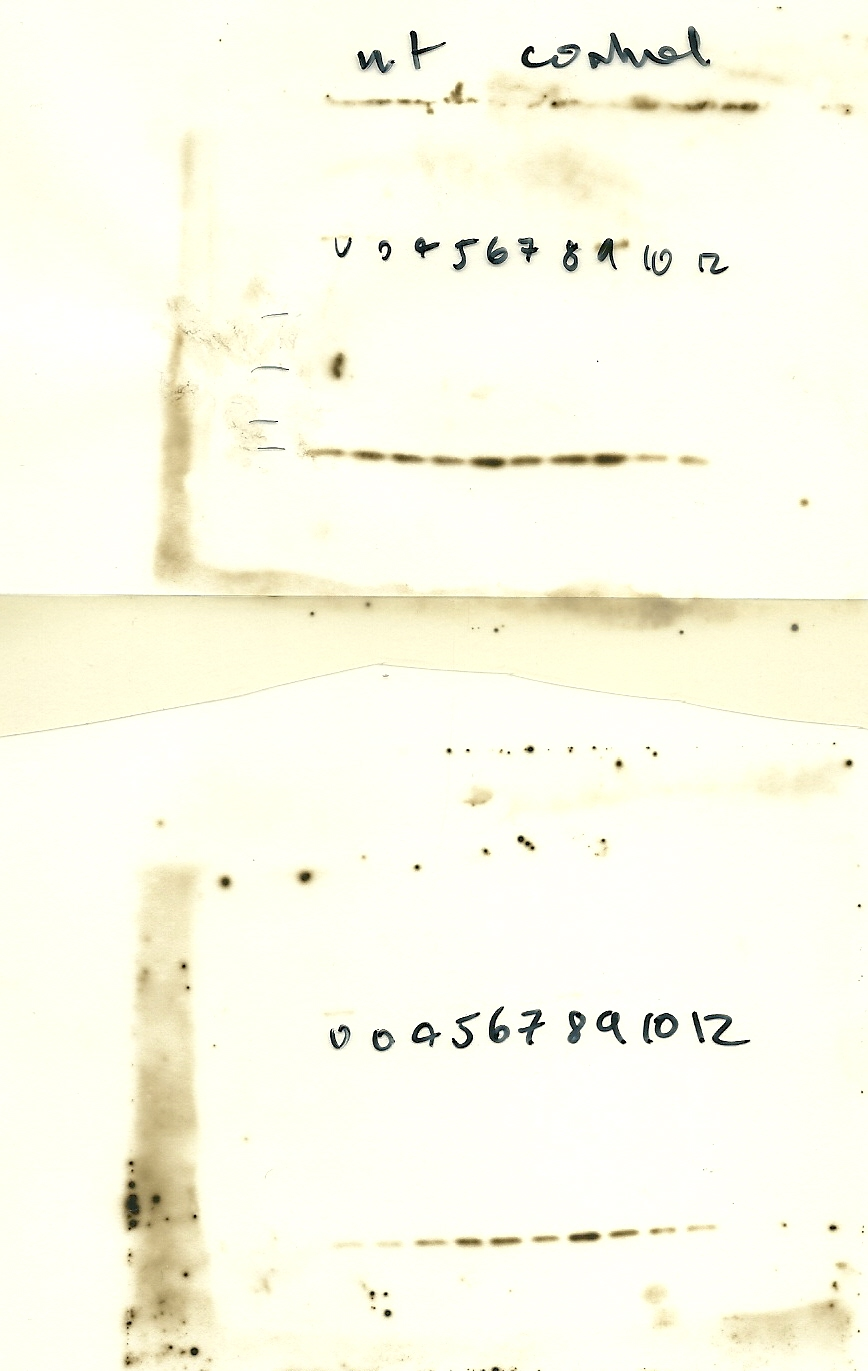

Supplement: Figure S1 — Raw blot 6B loading control (TIF) [file pone.0099688.s001.tif]

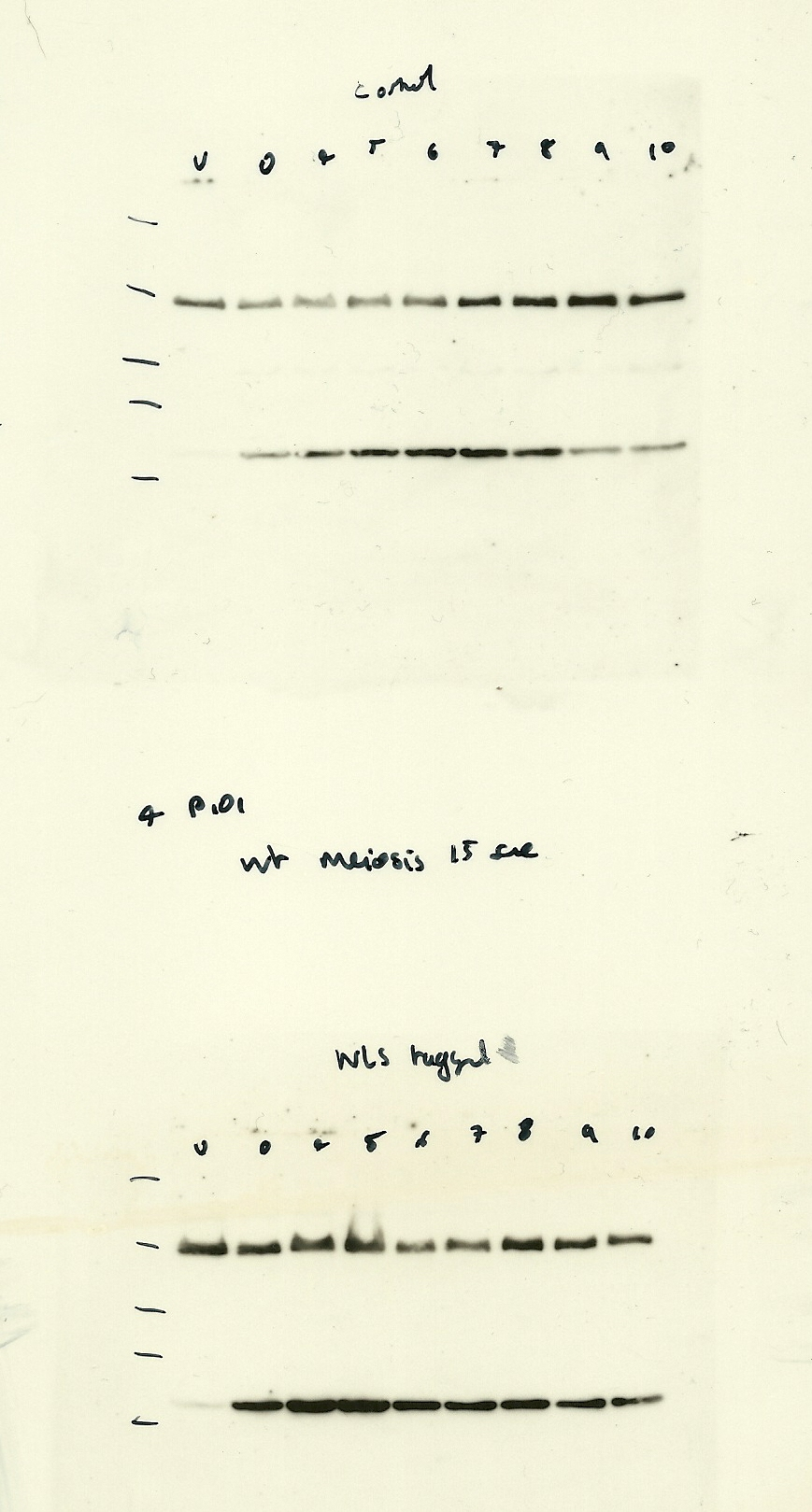

Supplement: Figure S2 — Raw blot 6C loading control (TIF) [file pone.0099688.s002.tif]

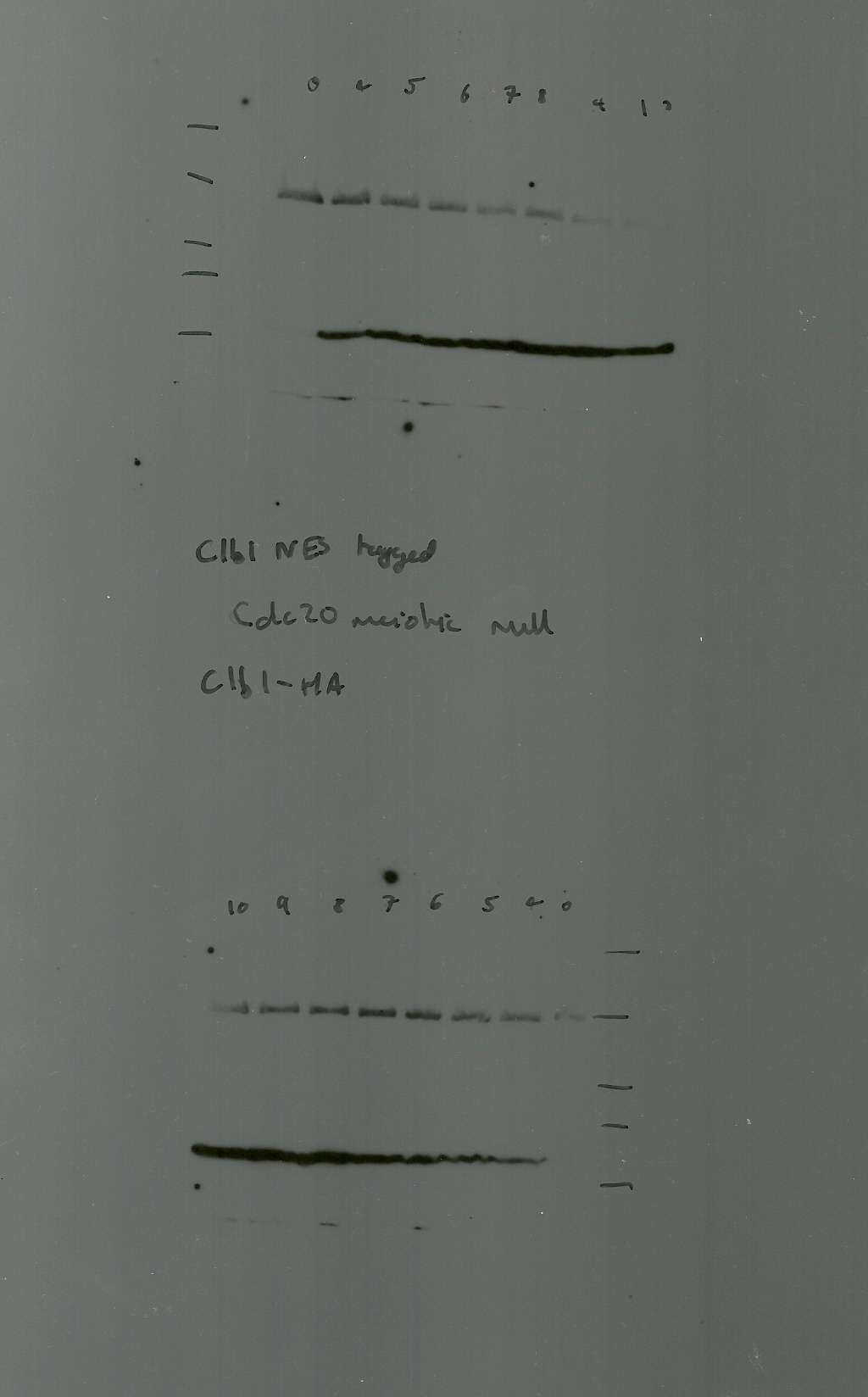

Supplement: Figure S3 — Raw blot 6D loading control (TIF) [file pone.0099688.s003.tif]

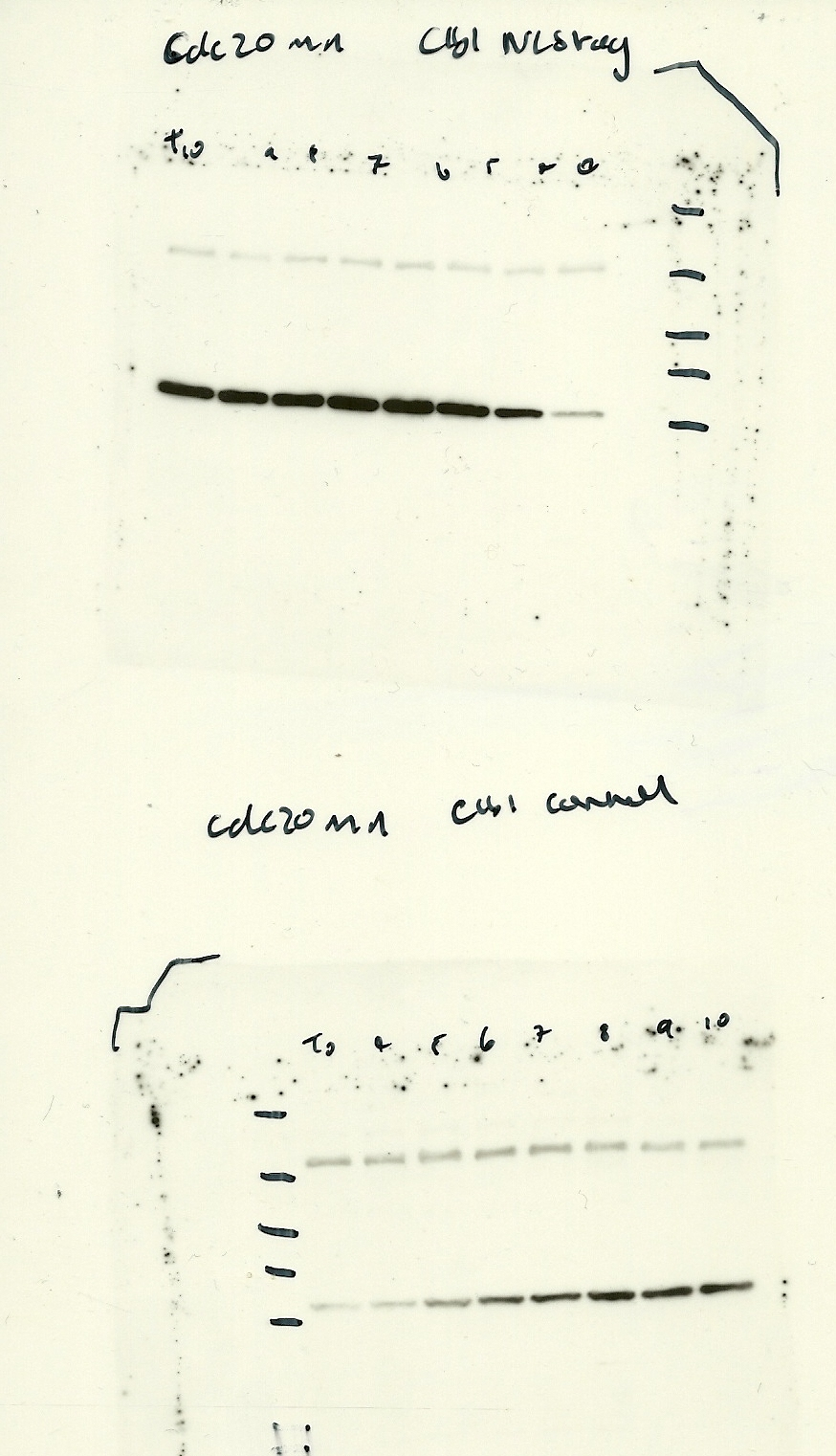

Supplement: Figure S4 — Raw blot 6E loading control (TIF) [file pone.0099688.s004.tif]

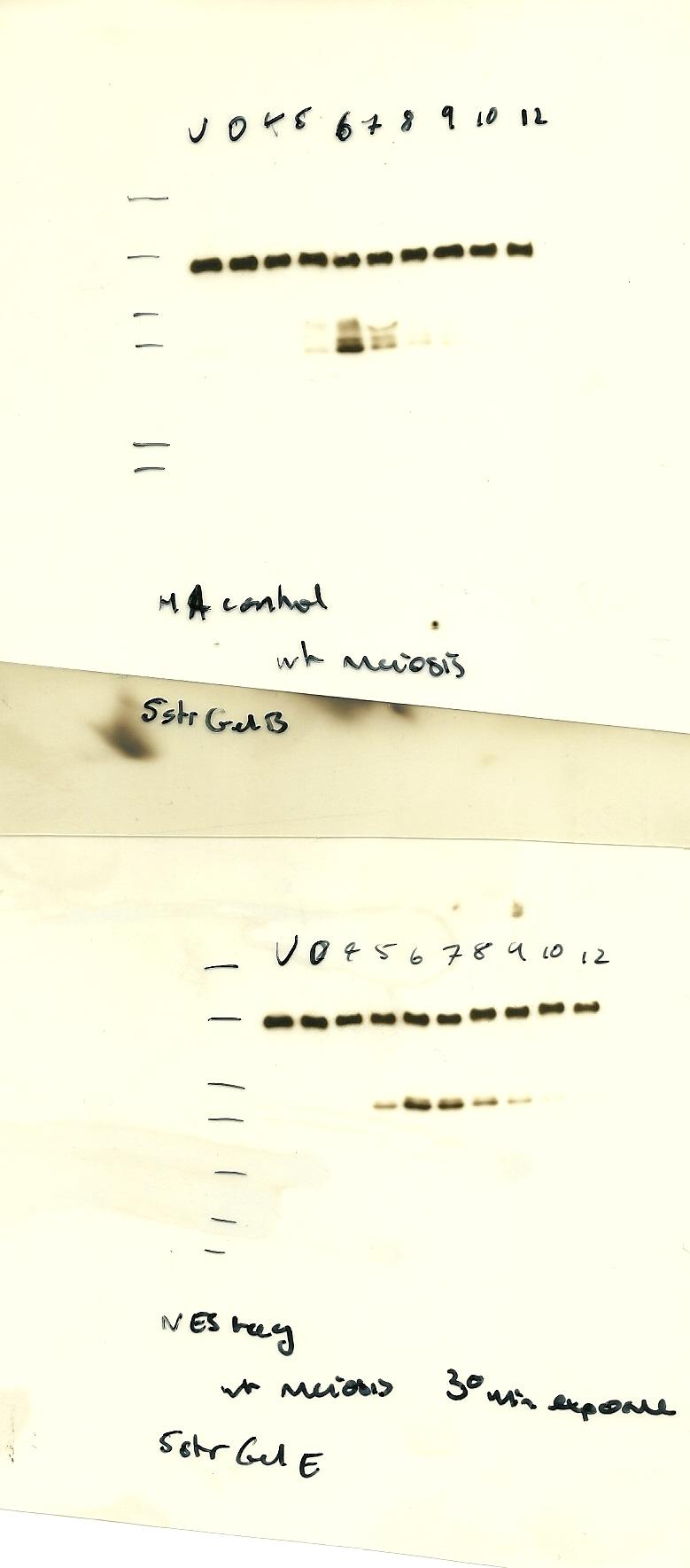

Supplement: Figure S5 — Raw blot 6B (TIF) [file pone.0099688.s005.tif]

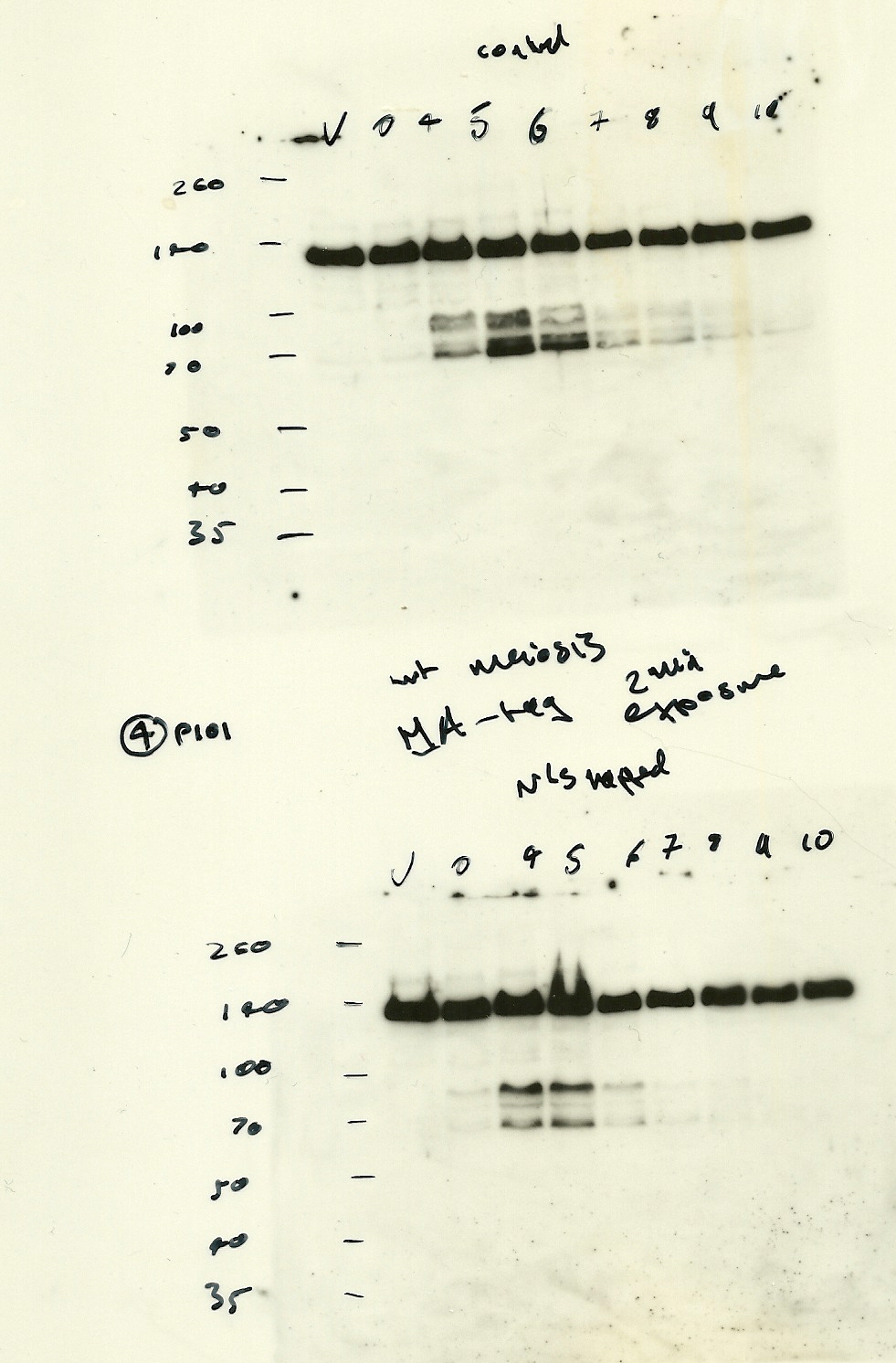

Supplement: Figure S6 — Raw blot 6C (TIF) [file pone.0099688.s006.tif]

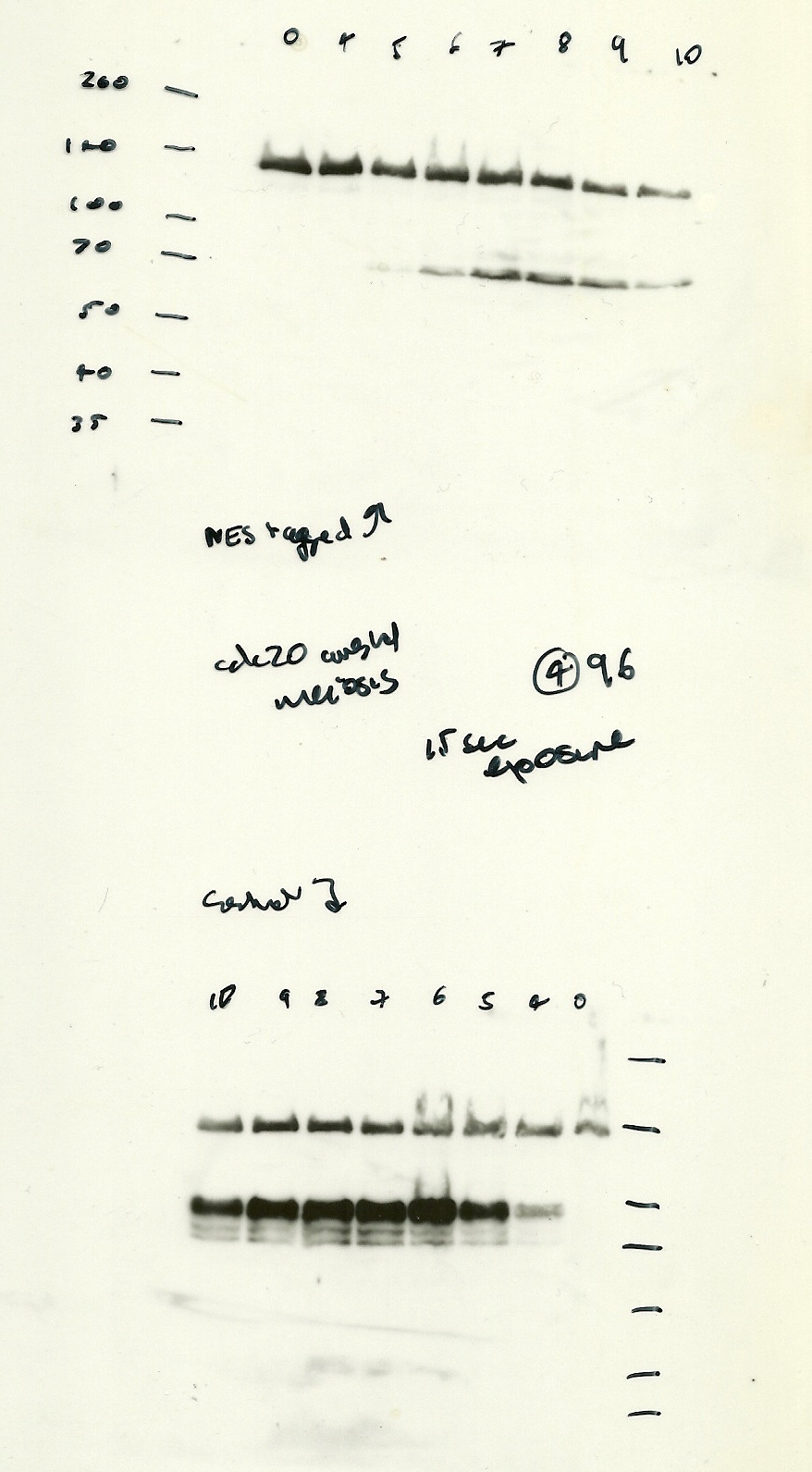

Supplement: Figure S7 — Raw blot 6D (TIF) [file pone.0099688.s007.tif]

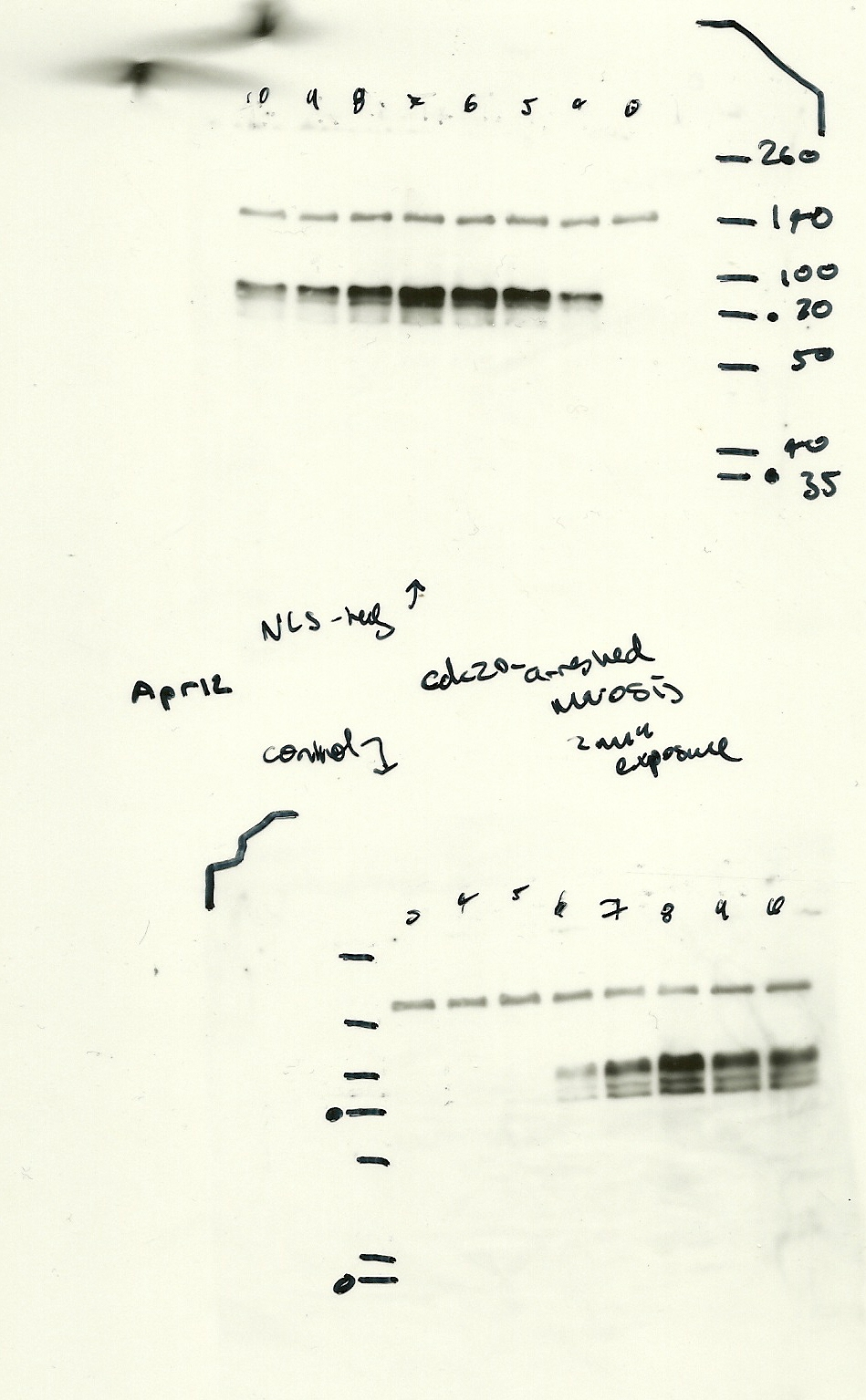

Supplement: Figure S8 — Raw blot 6E (TIF) [file pone.0099688.s008.tif]
